# Concurrent elevation of CO_2_, O_3_ and temperature severely affects oil quality and quantity in rapeseed

**DOI:** 10.1093/jxb/erw180

**Published:** 2016-05-23

**Authors:** Shahla Namazkar, Anders Stockmarr, Georg Frenck, Helge Egsgaard, Thilde Terkelsen, Teis Mikkelsen, Cathrine Heinz Ingvordsen, Rikke Bagger Jørgensen

**Affiliations:** ^1^Technical University of Denmark, DTU Environment, Risø Campus, Building 763, Frederiksborgvej 399, 4000 Roskilde, Denmark; ^2^Technical University of Denmark, Department of Applied Mathematics and Computer Science, Richard Petersens Plads, Building 324, 2800 Kongens Lyngby, Denmark; ^3^University of Innsbruck, Institute of Ecology, Sternwartestraße 15, 6020 Innsbruck, Austria; ^4^Danish Cancer Society, Statistics, Bioinformatics and Registry, Strandboulevarden 49, 2100 København Ø, Denmark; ^5^CSIRO, Plant Industry, Black Mountain Laboratories, GPO Box 1600, Canberra, ACT 2601, Australia

**Keywords:** *Brassica napus* L., greenhouse gases, model, multifactor experiment, oilseed rape, vegetable oil quality.

## Abstract

Plant oil is an essential dietary and bio-energy resource. Despite this, the effects of climate change on plant oil quality remain to be elucidated. The present study is the first to show changes in oil quality and quantity of four rapeseed cultivars in climate scenarios with elevated [CO_2_], [O_3_] and temperature (T) combined and as single factors. The combination of environmental factors resembled IPCC’s ‘business as usual’ emission scenario predicted for late this century. Generally, the climate scenarios reduced the average amounts of the six fatty acids (FAs) analysed, though in some treatments single FAs remained unchanged or even increased. Most reduced was the FA essential for human nutrition, C18:3-ω3, which decreased by 39% and 45% in the combined scenarios with elevated [CO_2_]+T+[O_3_] and [CO_2_]+T, respectively. Average oil content decreased 3–17%. When [CO_2_] and T were elevated concurrently, the seed biomass was reduced by half, doubling the losses in FAs and oil content. This corresponded to a 58% reduction in the oil yield per hectare, and C18:3-ω3 decreased by 77%. Furthermore, the polyunsaturated FAs were significantly decreased. The results indicate undesirable consequences for production and health benefits of rapeseed oil with future climate change. The results also showed strong interactive effects of CO_2_, T and O_3_ on oil quality, demonstrating why prediction of climate effects requires experiments with combined factors and should not be based on extrapolation from single factor experiments.

## Highlights

Oil quality in four rapeseed cultivars was severely impaired in a climate with simultaneous elevation of temperature, [CO_2_] and [O_3_]. The effects were unforeseeable from single factor experiments.

## Introduction

Current trends in greenhouse gas emissions suggest that the climate will change substantially in the 21st century (IPCC, 2013). Recent reports (Le Quéré *et al.*, 2014) indicate that the concentrations of greenhouse gases (CO_2_ equivalents) are still increasing – 2.3% in 2013 – and in the first half of 2015 the global average concentration of CO_2_ exceeded 400ppm (http://www.esrl.noaa.gov/gmd/ccgg/trends/). Current emissions are just above the RCP8.5 scenario, demanding substantial and likely unrealistic reductions in emissions by 2020 in order to keep the average global warming below 2 °C (IPCC, 2013).

The changing climate will affect global food security (IPCC, 2014). A number of studies have investigated crop productivity, e.g. in rapeseed (Supit *et al.*, 2010; Frenck *et al.*, 2011; Frenck *et al.*, 2013), and these show that production is likely to be seriously impeded if greenhouse gas emissions continue at the current pace. We previously showed that when [CO_2_] (~650±50ppm) and temperature (+5 °C above ambient) were elevated simultaneously, seed production was only 50% of that in rapeseed grown at ambient conditions (Frenck *et al.*, 2013). Though climate change effects on crop production are well researched, this is not the case for food quality. The recent IPCC report (AR5, Porter *et al.*, 2014) points out these uncertainties: ‘Little is known about combined effects of climate change factors on food quality or the economic and behavioral changes that will occur. Thus, there is little confidence regarding effects of climate change on human health through changes in nutrient composition’.

*Brassica napus* L., rapeseed or oilseed rape, is the second largest source of vegetable oil after soybean worldwide (USDA, 2016) and the predominant oil crop in Europe (European Commission, 2013). Rapeseed oil is being used in many parts of the world for food (canola oil) or bio-fuel, and additionally rapeseed meal is used for animal feed (McKevith, 2005; Wittkop *et al.*, 2009; Hoekman *et al.*, 2012). Despite its importance only a few studies have investigated the oil content and oil quality of rapeseed and related species under predicted future climate change conditions (Triboi-Blondel and Renard, 1999; Tripathi and Agrawal, 2012; Baux *et al.*, 2013), and treatments with combined climate factors have not been explored experimentally. The present study reports the effects of predicted future climate conditions on the quantity and quality of the seed oil from rapeseed. The results on changes in oil quantity and quality are viewed in the perspective of the decrease in seed yield observed under future changed climate conditions; seed yield was accounted for previously by Frenck *et al.* (2013). We applied treatments where [CO_2_], [O_3_] and temperature (T) were elevated one by one, and treatments where the factors were elevated concurrently. The combined scenarios mostly resembled conditions of RCP8.5 (IPCC, 2013), or A1FI (IPCC, 2007) around the years 2075–2100. With a continued increase in greenhouse gas emissions, the combination of 650ppm [CO_2_] and +5 °C applied in this study resembles a realistic future scenario.

Our study is the first to document the effects on the crop quality – specifically oil quality – from multifactor climate scenarios. The results suggests that reliable projections of the future quality of rapeseed oil should be derived from such multifactor studies and not made by assuming additivity of results from single factor experiments.

## Materials and methods

### Plant material

The fatty acids (FA) and total oil (lipid) content were analysed in mature seeds from rapeseeds cultivated during their entire life cycle in a climate phytotron under six different climate treatments. Four cultivars, ‘Bolero’, ‘Mary’, ‘Mozart’ and ‘Tanto’, were included in the study. The four cultivars were assumed to be representative for rapeseed, as they differed in year of release and breeding organization and covered different cultivation areas. All four cultivars belong to the ‘double low’ type (developed since the late 1970s), which was bred for low content of glucosinolates and erucic acid. Frenck *et al.* (2011) described the cultivars in more detail. Each cultivar was represented by an experimental population of 36 plants in each of the six climate treatments (216 plants per cultivar in total). Plants were cultivated at a density of 64 plants m^–2^ corresponding to a normal field density of 40–80 plants m^–2^. At maturity seeds were harvested and yield was determined. The quantity and quality of the seed oil were analysed from five replicate samples of the pooled seeds per cultivar-population and treatment. In total 120 seed samples were analysed (4 cultivars × 6 treatments × 5 replicates) for their content of FAs and total oil. After harvest the seeds were stored at 7 °C until sample preparation.

### Climate treatments in the phytotron

In the different treatments the cultivars were grown together but on separate wheeled cultivation tables. The six different treatments corresponded to six phytotron chambers each 24 m^2^ located at the Technical University of Denmark, DTU Risø Campus. Set points for the six different environments were:

- ambient – 390ppm [CO_2_], 19/12 °C (day/night), 20/20 ppb [O_3_] (day/night)
- all factors elevated – 650ppm [CO_2_], 24/17 °C, 60/20 ppb [O_3_]
- [CO_2_] and T elevated in combination – 650ppm [CO_2_], 24/17 °C, 20/20 ppb [O_3_]
- [CO_2_] elevated as single factor – 650ppm [CO_2_], 19/12 °C, 20/20 ppb [O_3_]
- T elevated as single factor – 390ppm [CO_2_], 24/17 °C, 20/20 ppb [O_3_]
- [O_3_] elevated as single factor – 390ppm [CO_2_], 19/12 °C, 60/20 ppb [O_3_].

Over the experiment, the average values for the manipulated environmental factors were as follows: ambient [CO_2_] 390±17ppm, elevated [CO_2_] 654±46ppm, ambient [O_3_] 18±16 ppb, elevated [O_3_] 52±11 ppb, ambient T 18.5±0.9/12.8±0.9 °C (day/night) and elevated T 23.3±0.8/17.9±0.8 °C (day/night). More information about growth conditions and phytotron treatments is given by Frenck *et al.* (2011, 2013).

Due to a limited number of the large phytotron chambers available, the two-factor combinations with elevated [O_3_] and elevated [CO_2_] or elevated T (O_3_+CO_2_ and O_3_+T) could not be executed; however, the modeling (see below) partly compensated for the incomplete design. Plants and their corresponding treatments were rotated both between and within the six chambers of the phytotron every week to minimize chamber effects (Frenck *et al.*, 2013).

During daytime (16/8h day/night light regime), photosynthetically active radiation (PAR) just above the canopy was 520 µmol photons m^−2^ s^–1^. Plants were watered by a drip irrigation system providing 4.4L m^−2^ day^−1^ delivered to the individual pots at the beginning of the daytime period. A relative humidity of 55/70% (day/night) was established in the growth chambers. All treatments received the same amount of water, and it could therefore be anticipated that treatments with elevated temperature experienced limitation of water to a higher degree than those with ambient temperature due to higher evapotranspiration. In treatments with elevated [CO_2_], the reduced stomatal conduction might partly mitigate the transpiration loss. However, the water deficit was not quantified.

### Analysis of FA composition and lipid content

#### Preparation of samples

Five replicates of 100mg seeds of each of the four rapeseed cultivars, times six treatments, were prepared and analysed in duplicate by gas chromatograph flame ionization chromatography using flame ionization detection (GC-FID) (Agilent Technologies 7890A). The seeds were ball-milled to obtain homogeneous samples (Retsch MM301 mixermill, Germany, stainless steel balls, d=20mm, 35mL grinding jar) for 10s. The sample size of 100mg seeds corresponded to approximately 26 seeds (average seed weight 3.9mg) randomly collected from the pooled seeds per cultivar and treatment. An amount of 5mL of chloroform/methanol mixture (2:1 v/v) together with 10mg internal standard (IS, C19:0) were added to each sample of crushed seeds. After evaporating the solvents, diethyl ether (4mL) was added to each extracted seed sample followed by addition of methanol (15mL). FA methyl esters (FAMEs) were prepared based on the methodology according to Craig and Murty (1959) by adding 25% sodium methoxide solution in methanol (1mL) to the extracted oil and maintaining this mixture at room temperature for 1h. After the reaction with sodium methoxide, cyclohexane (3mL) and distilled water (2mL) were added. The diluted FAMEs sample (1 µL) was introduced into the gas chromatograph using a CTC auto-sampler (Agilent, Denmark) in split mode (1:25). The FAMEs were separated using a 0.32mm i.d. × 30 m WCOT fused silica column coated with VF-23ms at a thickness of 0.25 μm (Agilent, Denmark). The carrier gas was helium at a flow rate of 1.0ml min^–1^. Separation of the FAMEs was achieved using a temperature gradient from 70 to 250 °C at 10 °C min^–1^.

#### Calibration

A concentration of 1mg mL^–1^ of C19:0 (the internal standard, IS) in cyclohexane was prepared as well as a set of calibration standards containing 50mg of C16:0, C18:0, C18:1, C18:2, C18:3 and C20:1 in 50mL cyclohexane. The stock standard solutions were prepared by adding 1, 0.75, 0.5 and 0.25mL of each FA standard solution to 1mL of the IS and the solutions were stored in darkness at 4 °C. These standards were analysed by GC-FID in order to establish the retention time and response factors of each compound. The method was validated using certified sunflower oil (Supelco 4-7123) and oil extracted from a rapeseed standard BCR-447 (COLZA, EC-JRC-IRMM, Geel Belgium).

### Statistical analysis of effects under the climate treatments

To investigate potential interaction between the individual factors in the combined treatments, lipid content and individual FAs were analysed with a mixed effects model. The fixed effects were modeled with an incomplete three-way factorial design on the treatment types (ambient, [CO_2_], [O_3_] and T). The effect of cultivar was considered random; cultivar-level random effects were applied in order to obtain a general predictive model. To investigate cultivar specific responses, the mixed effects model was modified to a multiple regression model such that the cultivar effects were considered as fixed, and allowed to interact with the treatment types. Prior to analysis, content of the six different FAs and the total lipid content were ln transformed. The transformed content of each FA and the total lipid content were modeled separately. The applied mixed effects model may be described as:

$$\begin{array}{l} \text{ln}\left({\text{lipid content}}_{\text{i}}\right)=\alpha +\beta_{{\text{CO}}_{\text{2}}}{\text{CO}}_{2}+\beta_{\text{T}}\text{T}+\beta_{{\text{O}}_{3}}{\text{O}}_{3}\\ \;+\beta_{{\text{CO}}_{\text{2}}:\text{T}}{\text{CO}}_{2}:\text{T}+\beta_{{\text{CO}}_{\text{2}}:\text{T}:{\text{O}}_{3}}{\text{CO}}_{2}:\text{T}:{\text{O}}_{3}\\ \;+\eta Y_{\text{Accession}}+\varepsilon_{i},\;i=\text{1},\;\ldots \;,\text{12}0 \end{array}$$ (1)

where the treatment symbols are indicators of whether the given treatment is part of the total treatment for the *i* ^th^ trial. So, for example, if the treatment in the *i* ^th^ trial consisted of CO_2_ alone, the indicator CO_2_ would be 1, while the remaining indicators for T and O_3_ and interactions would be equal to 0. The reference treatment is ambient, so that an ambient treatment will have all indicators equal to 0. *Y* indicates the random effect of *accession*. Because of the incompleteness of the factorial design, caused by the fact that combination treatments CO_2_+O_3_ and T+O_3_ were not included in the study, the third order interaction CO_2_:T:O_3_, and the two two-way interactions CO_2_:O_3_ and T:O_3_ could not be distinguished from each other. For this reason, an additive effect of the O_3_ treatment could only be tested versus the interaction effect with the simultaneous effect of CO_2_ and T. Thus, if an additive effect of the O_3_ treatment was rejected through testing the three-way interaction term CO_2_:T:O_3_, we could not say which of the factors – CO_2_ and/or T – interacted with O_3_. An additive effect of the CO_2_ and T treatments relative to each other was tested through the standard two-way interaction term CO_2_:T, as Eqn (1) without this term conforms with the additive effects.

Furthermore, a confirmatory analysis was carried out, where the content was analysed simultaneously for all FAs, with a model similar to Eqn (1). In this model, FA type was included as an explanatory variable, with main effects and interaction terms with the three basic treatment types. Confidence intervals are reported asymmetric, to reflect the non-linear ln transformation.

Model validation was performed through graphical procedures and normality tests of residuals. Parameter estimation was carried out using restricted maximum likelihood (REML), and tests were evaluated with T-distributions. All model analysis and testing were carried out using the software R, version 3.1.0 (R Core Team, 2014).

Lipid content (oil content) was calculated as the sum of the six individual FAs. Cultivar-specific variability of total oil and FA content to the different treatments is shown in Fig. 3. The figure was constructed by keeping the effect of cultivar fixed in Eqn (1), and allowing for interaction between cultivar, treatment types and fatty acid type. Standard deviations and significances are derived from Eqn (1). The total content of polyunsaturated fatty acids was also estimated with Eqn (1). Figure 4 depicts estimated changes relative to the ambient condition for total contents of polyunsaturated fatty acids, i.e. C18:2 and C18:3, according to Eqn (1).

## Results

**Fig. 1.**
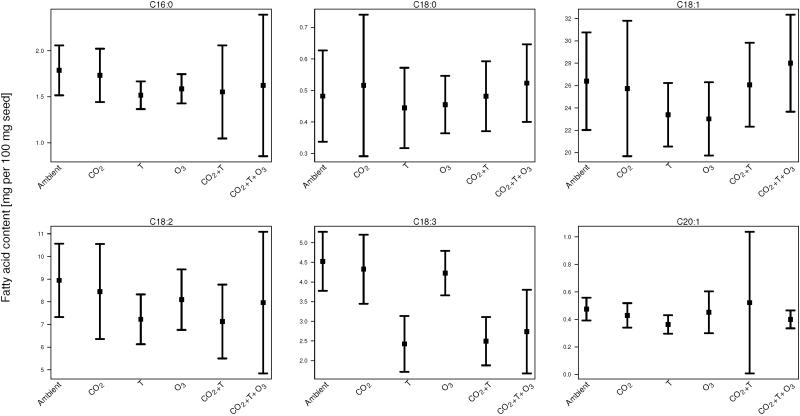
Summary of the effects of future climate scenarios, CO_2_, T, O_3_, CO_2_+T and CO_2_+T+O_3_, on the fatty acid content of rapeseed oil (mg fatty acid/100mg seeds). Mean±standard error for six fatty acids (average of the four cultivars).

**Fig. 2.**
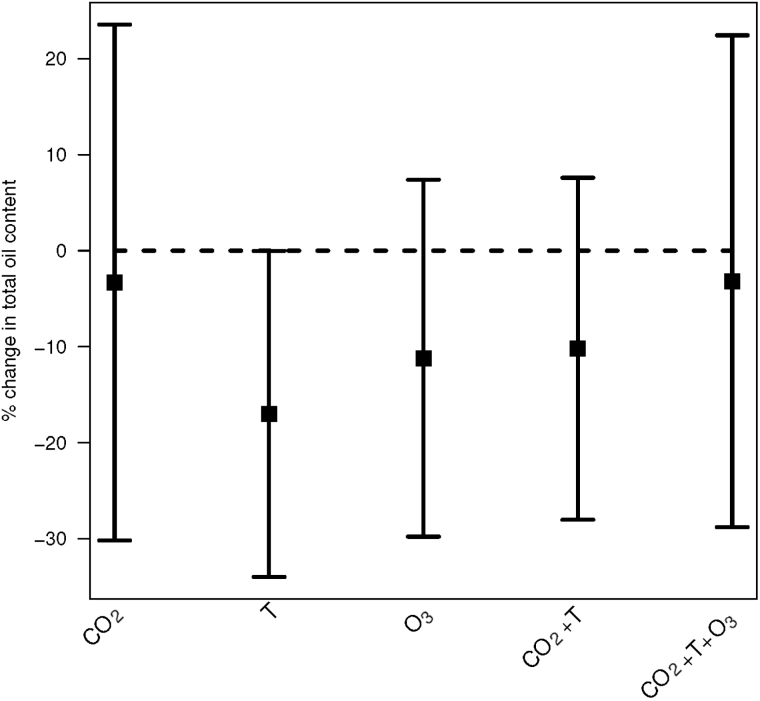
Change in total rapeseed oil content (average of four cultivars) in the climate treatment, CO_2_, T, O_3_, CO_2_+T and CO_2_+T+O_3_, relative to the ambient value (% change±standard error).

The contents of total lipid and FAs are given as percentage (%) of seed weight. Raw data can be found in Supplementary Table S1 at *JXB* online, and summaries of the close to 750 individual measurements of FAs and total oil are presented in Figs 1 and 2. The results show that the spectrum of FAs detected in the rapeseed samples was similar for all cultivars, and in agreement with previous reports on this crop (Omidi *et al.*, 2010; Tan *et al.*, 2011). Oleic acid (C18:1) was the predominant FA, while linoleic acid (C18:2) and linolenic acid (C18:3) were the second and the third most abundant FAs. Furthermore, palmitic (C16:0), stearic (C18:0), and eicosenoic acids (C20:1) were present in trace amounts in all rapeseed varieties. The quantification method of the FAs was specifically designed to detect the six most common FAs in rapeseed. However, other unexpected FAs could also be detected due to the high resolution of the chromatographic method.

The oil composition exhibited significant shifts in the amount and proportion of the individual FAs in the different treatments compared with ambient. The changes in FAs and lipid content averaged over the four cultivars are shown in Figs 1 and 2 and compared with ambient. The modeling (Table 1) revealed significant differences between treatments. It should be noted that the test statistics in Table 1 accounts for intra-correlation between treatment effect estimates. Therefore, overlapping confidence intervals for parameters do not necessarily translate to statistically insignificant differences.

**Table 1.** *Estimated levels of FAs and oil content and interaction between the factors CO*_*2*_ *, T and O*_*3*_ *(all according to Eqn (1*)). A *P*-value less than 0.05 for interaction between CO_2_ and T, and between (CO_2_,T) and O_3_ indicates that factors interact and additivity cannot be assumed.

FA	Treatment mg (100mg)^–1^ seeds, (95% confidence intervals), *P*-values for difference from ambient treatment						*P*-value for interaction between CO_2_ and T	*P*-value for interaction between (CO_2_,T) and O_3_
	Ambient	CO_2_	T	O_3_	CO_2_+T	CO_2_+T+O_3_		
C16:0	1.78 (1.69, 1.88) —	1.73 (1.63, 1.82) *P*=0.09	1.51 (1.43, 1.60) *P*<0.0001	1.58 (1.50, 1.67) *P*<0.0001	1.61 (1.52, 1.70) *P*<0.0001	1.70 (1.65, 1.75) *P*=0.01	0.0008	<0.0001
C18:0	0.47 (0.42, 0.53) —	0.51 (0.45, 0.57) *P*=0.005	0.44 (0.39, 0.50) *P*=0.008	0.45 (0.40, 0.51) *P*=0.18	0.48 (0.42, 0.54) *P*=0.91	0.52 (0.46, 0.59) *P*=0.005	0.58	0.009
C18:1	26.31 (24.52, 28.19) —	25.57 (23.83, 27.40) *P*=0.17	23.35 (21.76, 25.02) *P*<0.0001	22.70 (20.92, 24.58) *P*<0.0001	26.01 (24.25, 27.88) *P*=0.59	27.93 (26.02, 29.93) *P*=0.004	<0.0001	<0.0001
C18:2	8.82 (7.90, 9.83) —	8.49 (7.60, 9.46) *P*=0.03	7.30 (6.53, 8.13) *P*<0.0001	8.08 (7.22, 9.01) *P*=0.0002	7.02 (6.28, 7:82) *P*<0.0001	7.82 (6.99, 8.73) *P*<0.0001	0.22	<0.0001
C18:3	4.42 (4.05, 4.83) —	4.39 (4.02, 4.79) *P*=0.76	2.45 (2.24, 2.67) *P*<0.0001	4.22 (3.85, 4.62) *P*=0.15	2.43 (2.22, 2.65) *P*<0.0001	2.69 (2.45, 2.94) *P*<0.0001	0.11	0.004
C20:1	0.47 (0.44, 0.50) —	0.43 (0.40, 0.46) *P*<0.0001	0.36 (0.34, 0.39) *P*<0.0001	0.42 (0.40, 0.46) *P*<0.0001	0.41 (0.38, 0.44) *P*<0.0001	0.40 (0.37, 0.43) *P*<0.0001	<0.0001	0.02
Total oil (lipid sum)	42.49 (39.39, 45.78) —	41.26 (38.23, 44.45) *P*=0.08	34.51 (31.96, 37.21) *P*<0.0001	37.78 (35.02, 40.70) *P*<0.0001	38.22 (35.43, 41.17) *P*<0.0001	41.06 (38.06, 44.23) *P*=0.03	<0.0001	<0.0001

### Fatty acids

All applied climate scenarios affected FA content, though in some treatments not all FAs were affected, e.g. the treatment with elevated [CO_2_] only had effects on C18:0, C18:2 and C20:1. The T increase of +5 °C day/night was the single factor with the largest detrimental effect on FA content as well as the quantity of the seed oil (Table 1). All six FAs were significantly decreased by this treatment, 8–46% (on average; Fig. 1 and Supplementary Table S1). The ozone treatment affected C16:0, C18:1, C18:2 and C20:1 significantly (Table 1) with decreases of 5–13% (Fig. 1 and Supplementary Table S1).

For the climate treatments combining elevated [CO_2_], T and [O_3_], the FA changes recorded were predominantly unfavorable. In the scenario with all three factors elevated simultaneously, decreases were significant for the FAs C16:0 (9%), C18:2 (11%), C18:3 (39%) and C20:1 (16%), but the content of C18:0 and C18:1 increased (9% and 6%, respectively) (Table 1, Fig. 1 and Supplementary Table S1). When [CO_2_] and T were elevated concurrently but [O_3_] kept constant and low, reductions were significant for the FAs C16:0 (13%), C18:2 (20%) and C18:3 (45%) (Table 1, Fig. 1 and Supplementary Table S1).

The interactive effects between factors were modeled for the treatments with combination of factors. For the individual FAs, the interaction between elevated [CO_2_] and elevated T in their two-factor treatment was significant for C16:0, C18:1 and C20:1 (Table 1). The interaction between elevated [O_3_] and elevated [CO_2_]+T, (CO_2_,T), was found to be significant for all six FAs. For example, the estimated effect of the three-factor treatment on C18:3 was larger than could be expected from additivity of the three single factor treatment effects for this FA (*P*=0.004, Table 1).

### Total lipid

The modeling revealed that the future climate scenarios decreased the oil content (Table 1). The treatments with elevated [CO_2_] and T combined produced about 10% less lipid, and when all three factors were elevated simultaneously, the lipid sum decreased by 3%. In the single factor treatments with elevated T or O_3_, the lipid content was reduced by approximately 17% and 11%, respectively (Fig. 2 and Supplementary Table S1).

There was a significant interaction between [CO_2_] and T in their effect on total lipid (*P*<0.0001), and the effects from single factor treatments were not additive (Table 1). All three factors combined also had a greater effect than expected additive effects from single factor treatments. Assuming additivity of single factors, the expected lipid content would be 42.49–13.92=28.57. However, the expected lipid content according to Table 1 when combining all factors was 41.06, which is greater than the expectation when assuming additivity of the single factor effects (*P*<0.0001).

### Cultivar responses

**Fig. 3.**
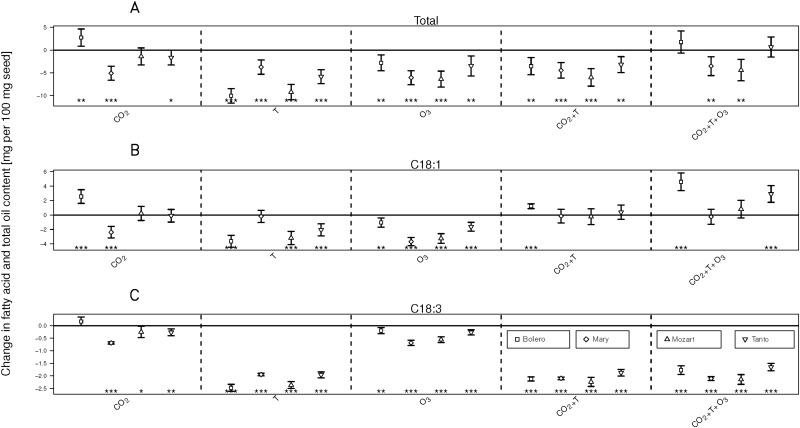
Cultivar-specific changes in total rapeseed oil (A), and the fatty acids C18:1 (B) and C18:3 (C) content in the climate treatments, CO_2_, T, O_3_, CO_2_+T and CO_2_+T+O_3_, relative to the ambient value (absolute change±standard error). Derived from Eqn (1) with cultivar effects kept fixed. Asterisks (***P<0.001, **P<0.01, *P<0.05) indicate cultivar-specific responses to the corresponding treatment.

The response of individual cultivars to the climate treatments are illustrated in Fig. 3 for total oil and two of the most important FAs from rapeseed, C18:3 and C18:1. Significant differences in the response of cultivars were noted for total oil across all treatments – the general trend was a decrease in oil content. Cultivars also showed different responses in C18:1 content under different climate treatments. Significant reductions in content were most common, but also significant increases in content were observed for the cultivars Bolero and Tanto, when they were grown in elevated [CO_2_] or the three-factor treatment. Also for FA C18:3 differences among cultivars were observed in all treatments. All cultivars presented a significant reduction in C18:3 except Bolero, which showed no change in C18:3 content in elevated [CO_2_]. Also for the other FAs (data not shown) variation among cultivars was the rule.

### Change in fraction of the polyunsaturated FAs in the seed oil

**Fig. 4.**
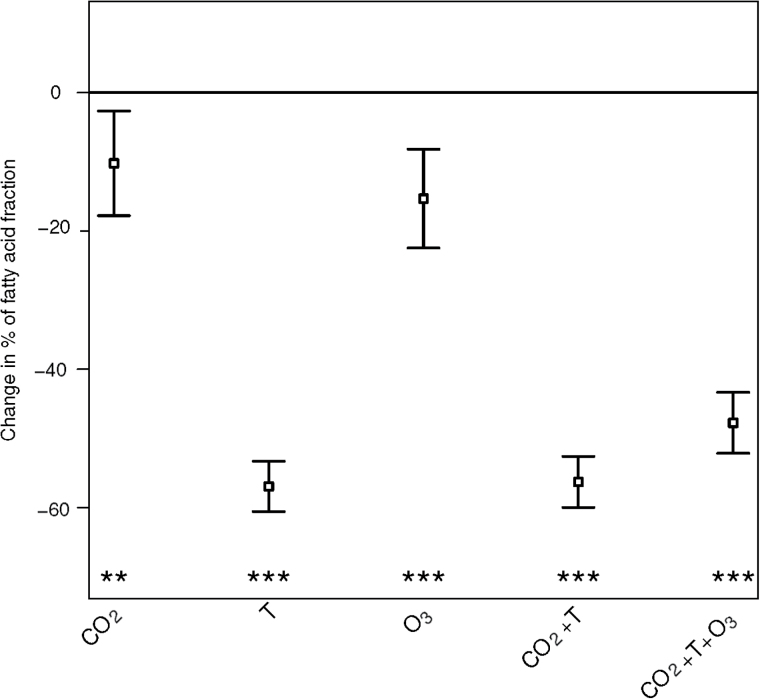
Change in polyunsaturated fatty acid fraction of rapeseed oil in the climate treatments, CO_2_, T, O_3_, CO_2_+T and CO_2_+T+O_3_, relative to the ambient value (% change±standard error). The polyunsaturated fatty acids were C18:2 and C18:3. Derived from Eqn (1). (***P<0.0001, **P<0.01, *P<0.05.)

In the present study the polyunsaturated FAs – considered an especially healthy component of the diet – were found to be significantly decreased by the climate treatments. The fraction of the two polyunsaturated FAs, C18:2 and C18:3 (ω3, omega 3) of the total seed oil was reduced in all treatments relative to ambient (Fig. 4).

## Discussion

The effect of multiple aspects of climate change on food quality has only been documented by a few experimental studies. Little is known about the effects of combining climate factors, making multi-treatment experiments and modeling pivotal; however, the single factor treatments are also needed for a fundamental understanding of the mechanisms of response.

### Effects of the single factor treatments

#### Elevated [CO_2_]

Several authors (e.g. Long *et al.*, 2006; Hatfield *et al.*, 2011; IPCC 2014) have reviewed effects of the changing climate on crop production. It is generally assumed that elevated [CO_2_] will increase crop production (Hatfield *et al.*, 2011). Many studies have reported that plants benefit from elevated [CO_2_] due to a boost in photosynthesis, decreased stomatal conductance and improved leaf water status resulting in increased productivity (Leakey *et al.*, 2009; Högy *et al.*, 2010; Wang *et al.*, 2012). When it comes to the quality of oil crops, the limited research available indicates that elevated [CO_2_] may have a positive effect, but certainly there are also negative impacts on oil quality by changing the composition of FAs on a dry weight basis. Illustrated by the handful of studies available on a very limited number of cultivars in sunflower (Pal *et al.*, 2014), castor bean (Vanaja *et al.*, 2008) and rapeseed (Högy *et al.*, 2010; Tian *et al.*, 2014), the crop quality response to elevated [CO_2_] is not unequivocal. A growth chamber study of the rapeseed cultivar ‘Mozart’ – likely the same cultivar also analysed in the present study – demonstrated decreased seed oil% as well as seed yield, while vegetative biomass increased (Franzaring *et al.*, 2011; [CO_2_] 550ppm). Averaged over all four cultivars of rapeseed our results showed that [CO_2_] enrichment did not affect the oil% significantly; C18:0 increased and C18:2 and C20:1 were reduced (Table 1, Figs 1 and 2 and Supplementary Table S1). When comparing the response of individual cultivars, significant differences were noted for elevated [CO_2_] (Fig. 3); however, ‘Mozart’ did not have changed oil% (Fig. 3), seed yield and aboveground biomass (Frenck *et al.*, 2011), results that are contrary to the observations by Franzaring *et al.* (2011). The fact that the results on oil quality from elevated [CO_2_] are not in agreement very likely reflects both inter- and intraspecific variation as well as experimental conditions. For example, the level of [CO_2_] in the experiments will affect the photosynthetic response, transpiration, as well as the nutrients available (Franzaring *et al.*, 2011). This variability among studies underlines the need for analysis of more and diverse genotypes of a crop species grown in different environmental settings to create a broad spectrum of responses.

One suggested mechanism responsible for changes in FA content under elevated [CO_2_] is that by increasing the availability of carbon, the production of malonyl CoA (the precursor required for synthesis of long- and short-chain FAs) changes, which possibly causes imbalance in FA proportions (Chakraborty and Uprety, 2012).

#### Elevated temperature

In the present study elevated temperature was the factor affecting the oil composition and total oil the most. The lipid sum as well as all FAs were significantly decreased at a temperature increase of 5 °C (Table 1), and particularly the change in polyunsaturated FAs was large in treatments where temperature was elevated (Fig. 4). The effects of elevated T will certainly depend on the magnitude of the treatment and the cardinal temperature of the species. In the RCP8.5 scenario with a possible 4–5 °C elevation of temperature, global crop yields are expected to decrease (Porter *et al.*, 2014). However, few experimental studies have analysed the quality of crops from scenarios where temperature is increased by 4 °C or more over the entire growing period (Porter *et al.*, 2014). Werteker *et al.* (2010) studied the effect of temperature on oil quality in rapeseed, soybean and sunflower in the last month before harvest. They found that in general, there was a negative correlation between temperature and the proportion of C18:3 and C18:2 in the crop oil. The same negative effect of elevated temperature on C18:3 has been reported in other studies (Thomas *et al.*, 2003; Kumar *et al.*, 2006). Byfield and Upchurch (2007) reported that higher temperatures increased expression of ω-3 FA desaturase in soybean, possibly resulting in lower C18:3 and higher C18:0 concentrations. In accordance with this, our treatments with elevated T significantly reduced concentrations of C18:3. In contrast C18:0 was found to be reduced in our study. The correct ratio of FAs in the human diet is important, especially because C18:3 is an essential FA that humans cannot synthesize.

#### Elevated ozone

Effects of elevated ozone will depend on the regional tropospheric concentration and also on the tolerance of crop species to the oxidative stress (Mills *et al.*, 2007). The yield losses due to elevated [O_3_] are estimated to be 10% for wheat and soybean and 3–5% for maize and rice (Van Dingenen *et al.*, 2009) since preindustrial times. In the present study it was found that the general effect of elevated [O_3_] was a decrease in total lipid content, and a significant reduction in the content of the FAs C16:0, C18:1, C18:2 and C20:1, whereas C18:0 and C18:3 were unaffected (Table 1, Figs 1 and 2 and Supplementary Table S1). Also a previous report (De Bock *et al.*, 2011) found a reduction in lipid content of rapeseed, when ozone was increased 8 hours per day by 20 or 40 ppb above an ambient ozone concentration of 29–33 ppb. The authors suggested that this observation was explained by a change in carbon partitioning with an increased allocation of carbon to leaf injury repair. Tripathi and Agrawal (2013) exposed two cultivars of linseed to elevated [O_3_] alone (27.7–59.0 ppb (ambient) + 10 ppb) and in combination with UV-B. They found that both as an individual factor and in combination with UV-B, ozone reduced oil content and oil quality of linseed; rancid oil of low nutritional value was favored. The same authors (Tripathi and Agrawal, 2012) analysed the effects of elevated [O_3_] (26.3–69.5 ppb (ambient) + 10 ppb) in two cultivars of *Brassica campestris*, one of the progenitor species of oilseed rape. In this species, they found an increase in C18:1 and C18:3.

### Interactive effects of factors in two- and three-factor treatments

The plant phenotypes resulting from an environment with a simultaneous increase of multiple factors like elevated [CO_2_], T and [O_3_] are little studied and not well understood. Generally, the effect of elevated T and elevated [O_3_] on plant production are considered counteractive to the effect of elevated [CO_2_] (Tianhong *et al.*, 2005; Ainsworth *et al.*, 2008; Taub *et al.*, 2008; Gillespie *et al.*, 2012, Long, 2012). Our results on crop quality are consistent with this finding (Table 1), but additionally our analysis indicates that the effects of factors in combination cannot be estimated from combining results of single factor experiments. For example, in the scenario where T and CO_2_ interacted, it was found that the content of C16:0, C18:1 and C20:1 could not have been predicted from the results of the individual single factor treatments (Table 1, second to last column). Also for the lipid content the outcome from combining T and CO_2_ was influenced by their interaction (Table 1, second to last column). If [O_3_] was increased together with T and [CO_2_] in the three-factor combination, O_3_ interacted with (CO_2_,T) and influenced all FAs and the collective lipid sum (Table 1, last column). Undoubtedly the more interacting factors present in the environment, the more uncertainty there will be in extrapolating results from single factor experiments. Likewise, with respect to the response of individual cultivars in climate scenarios with combined factors, results from single factor treatments might not be additive (Fig. 3). The interactive effect of O_3_ and CO_2_ has been studied previously in regard to yield and oil content of *Brassica juncea* (Indian mustard), and the oil content was found to decrease at elevated [O_3_] (25–35 ppb). This reduction was only partly compensated by exposing the plant to elevated [CO_2_] (550ppm [CO_2_]; Singh *et al.*, 2013). Unfortunately, we were not able to resolve if O_3_ interacted with CO_2_, T or both factors at the same time, as the limitation of growth chambers prevented experimentation of the two-way interactions CO_2_:O_3_ and T:O_3_.

### Combined effects of reductions in seed yield, oil quantity and quality

Besides the unfavorable effects on oil content and quality of rapeseed, a reduction in seed production of this crop can be foreseen in a changed climate (Frenck et al., 2011, 2013; Supplementary Table S2). Compared with ambient conditions, the treatment with elevated [CO_2_] did not increase seed production significantly, while under elevated T and the combination of elevated [CO_2_] and T, the seed yield was decreased by approximately 50% (Frenck *et al.*, 2013; Supplementary Table S2). This trend in seed production is supported by previous studies on wheat and rapeseed, where growth was studied in the two-factor treatments with elevated [CO_2_] and T (Qaderi *et al.*, 2006; Manoj-Kumar *et al.*, 2012). In the two-factor treatment with elevated [CO_2_] and T in our study, the essential FA, C18:3, decreased by 45% in addition to the 50% reduction of the seed set, and the oil content decreased by 10%. We acknowledge that our results were obtained in a phytotron, and therefore comparable experiments with combinations of elevated [CO_2_] and T in the field might not have the same outcome. However, such experiments have not yet been performed because of technical and economic challenges. Modeling of European crop yields under climate change conditions has been performed (Supit *et al.*, 2012), and the results indicated that in northern Europe yields of spring crops either stagnate or decline depending on the magnitude of increase in T and [CO_2_]. If the results obtained in our study reflect a future worst case scenario, climate change effects on spring oilseed rape could be profound. Currently the average seed yield of spring rapeseed in Denmark is 2.48 ton ha^–1^ (StatBank Denmark, 2016). However, assuming a yield reduction of 50%, when [CO_2_] and T are elevated simultaneously to levels applied in the present study, the seed harvest will be reduced to 1.24 ton ha^–1^. The oil yield will drop from the present 1.12 ton ha^–1^ (45 wt% oil/seed; Wittkop *et al.*, 2009) to 0.47 ton ha^–1^ (38 wt% oil/seed in our two-factor treatment), and the content of C18:3 will fall from 0.11 ton ha^–1^ (today C18:3 constitutes approximately 10wt% of the oil (Omidi *et al.*, 2010); in the future the content will be reduced by 45%) to 0.03 ton ha^–1^, when reductions in seed weight, oil content and C18:3 are added up. If this worst case scenario becomes a reality, the harvested oil per hectare will be reduced by 58%, and content of C18:3 will decrease by 77%. For the top three rapeseed producing countries, Canada (rapeseed crop worth approximately 8 billion CAD), India and China (each with a rapeseed area of 7–8 Mha) such a reduction would have significant economic consequences.

### Unsaturated FAs

Unsaturated FAs are preferred in the human diet (McKevith, 2005; Gebauer *et al.*, 2006), since they are assumed to reduce the risk of cardiovascular diseases, while for bio-fuel storage saturated FAs are favored, as a decline in saturation causes an increase in oxidation of FA and therefore less stability (Hoekman *et al.*, 2012). Especially the polyunsaturated FAs are assumed to have health benefits (Kris-Etherton *et al.*, 2004; Reed *et al.*, 2007). Therefore, the consequences for human nutritional value of the observed dramatic decrease in C18:2 plus C18:3 (Fig. 4) in combinations of elevated [CO_2_], T and [O_3_] are profound, and could be a challenge for the future quality of rapeseed oil for consumption.

Though C18:1 is a monounsaturated FA, it is the most abundant and preferred FA in vegetable oil for bio-fuel production (Pinzi *et al.*, 2013). This FA was not reduced by the two-factor treatment, and even somewhat increased in the three-factor treatment (Table 1, Fig. 1). Therefore, bio-fuel quality may not be negatively affected by the future climate changes. However, oil quantity per hectare will decrease and affect the harvested amount of any FA.

### Outlook

The overall decrease in quality and quantity of rapeseed oil found in the present study depicts a pessimistic future for seed oil production from rapeseed and for the health benefits of rapeseed oil. However, the difference in cultivar responses indicates that cultivars holding specific beneficial genes may be identified and exploited to retain oil quality and quantity despite comprehensive changes in the climate. At this point it seems advisable and profitable to initiate screening programs for genetic resources of crop plants in order to optimize food quality under future climate conditions.

## Supplementary data

Supplementary data are available at *JXB* online.

Table S1. Data set on contents of fatty acids and total lipid.

Table S2. Detailed data on seed yield.

Supplementary Data
