# Supplementary material for: Concurrent elevation of CO2, O3 and temperature severely affects oil quality and quantity in rapeseed
Source: J Exp Bot. 2016 May 23;67(14):4117–25. doi: 10.1093/jxb/erw180 (PMC5301921; doi:10.1093/jxb/erw180)
Supplement: Supplementary Data [file supp_67_14_4117__index.html]

Concurrent elevation of CO2, O3 and temperature severely affects oil quality and quantity in rapeseed — Concurrent elevation of CO2, O3 and temperature severely affects oil quality and quantity in rapeseed — Supplementary Data 

# Concurrent elevation of CO2, O3 and temperature severely affects oil quality and quantity in rapeseed

## Supplementary Data

Data files

- supplementary\_tables\_S1\_S2.xlsx - Supplementary Data
